# Integrated analysis of the whole transcriptome of skeletal muscle reveals the ceRNA regulatory network related to the formation of muscle fibers in Tan sheep

**DOI:** 10.3389/fgene.2022.991606

**Published:** 2022-10-18

**Authors:** Ran Cui, Xiaolong Kang, Yufang Liu, Ximing Liu, Shuheng Chan, Yubei Wang, Zhen Li, Yao Ling, Dengzhen Feng, Menghua Li, Fenghua Lv, Meiying Fang

**Affiliations:** ^1^ Department of Animal Genetics and Breeding, National Engineering Laboratory for Animal Breeding, MOA Laboratory of Animal Genetics and Breeding, College of Animal Science and Technology, China Agricultural University, Beijing, China; ^2^ College of Agriculture, Ningxia University, Yinchuan, China; ^3^ Key Laboratory of Animal Genetics, Breeding and Reproduction of Ministry of Agriculture and Rural Affairs, Institute of Animal Science, Chinese Academy of Agricultural Sciences, Beijing, China

**Keywords:** Tan sheep, skelete muscle, lncRNA, circRNA, ceRNA

## Abstract

Meat quality is highly influenced by the kind of muscle fiber, and it can be significantly improved by increasing the percentage of slow-twitch fibers. It is still not known which genes control the formation of muscle fibers or how those genes control the process of forming in sheep until now. In this study, we used high-throughput RNA sequencing to assess the expression profiles of coding and noncoding RNAs in muscle tissue of Tan sheep and Dorper sheep. To investigate the molecular processes involved in the formation of muscle fibers, we collected two different muscle tissues, *longissimus dorsi* and *biceps femoris*, from Tan sheep and Dorper sheep. The *longissimus dorsi* of Tan sheep and Dorper sheep displayed significantly differential expression levels for 214 lncRNAs, 25 mRNAs, 4 miRNAs, and 91 circRNAs. Similarly, 172 lncRNAs, 35 mRNAs, 12 miRNAs, and 95 circRNAs were differentially expressed in the *biceps femoris* of Tan sheep and Dorper sheep according to the expression profiling. GO and KEGG annotation revealed that these differentially expressed genes and noncoding RNAs were related to pathways of the formation of muscle fiber, such as the Ca^2+^, FoxO, and AMPK signaling pathways. Several key genes are involved in the formation of muscle fibers, including ACACB, ATP6V0A1, ASAH1, EFHB, MYL3, C1QTNF7, SFSWAP, and FBXL5. RT-qPCR verified that the expression patterns of randomly selected differentially expressed transcripts were highly consistent with those obtained by RNA sequencing. A total of 10 lncRNAs, 12 miRNAs, 20 circRNAs, and 19 genes formed lncRNA/circRNA-miRNA-gene networks, indicating that the formation of muscle fiber in Tan sheep is controlled by intricate regulatory networks of coding and noncoding genes. Our findings suggested that specific ceRNA subnetworks, such as circ_0017336-miR-23a-FBXL5, may be critical in the regulation of the development of muscle fibers, offering a valuable resource for future study of the development of muscle fibers in this animal species. The findings increase our understanding of the variety in how muscle fibers originate in various domestic animals and lay the groundwork for future research into new systems that regulate the development of muscle.

## Introduction

Meat quality is an important economic trait, and the ratio of muscle fibers is one of the most important factors affecting meat quality traits, which can be significantly improved by increasing the proportion of slow-twitch fibers. Skeletal muscle is composed of two types of muscle fibers that are classified as slow-twitch and fast-twitch fibers, which have different metabolic and contractile properties. Compared with fast-twitch fibers, slow-twitch fibers have higher oxidative metabolism capacity and higher mitochondrial content, so their proportions are positively correlated with various aspects of meat quality, such as tenderness, flavor, and juiciness (Doerr, 1971; RyuandB and Kim, 2005; Su et al., 2013). Ningxia Tan sheep are an important breed for fur in China (Kang et al., 2013; Liu et al., 2018), but with the decline of the fur market, they have gradually been bred for meat. Previous studies have shown that some meat quality indicators of Tan sheep were measured, such as pH, hydraulic power, meat color, shear force, cooked meat percentage, muscle fiber density, and diameter, while the relationship between the proportion of muscle fibers and meat quality traits in Tan sheep requires a more precise interpretation; in this study, we compared the proportions of slow-twitch fibers in Tan sheep and Dorper sheep. Studies have also found the heritability of the muscle fiber content ranged from 0.20 to 0.68 (Larzul et al., 1999), and molecular breeding is a key method used for the improvement of slow-twitch fiber content. However, no effective molecular markers for slow-twitch fiber content selection practices in sheep have yet been found.

A growing number of studies have revealed that noncoding RNAs are involved in transcriptional regulation (Kotb et al., 2015), cellular communication, and signal transduction (Kristensen et al., 2019) and play a variety of significant roles in muscle growth and development (Hong et al., 2019). Competing endogenous RNA (ceRNA) is a common physiological mechanism by which lncRNAs and circRNAs compete with miRNAs for binding (Salmena et al., 2011). Previous studies have demonstrated noncoding RNA and ceRNA regulation of myoblast differentiation and the formation of muscle fibers in mouse, chicken, and bovine species. For example, the intronic lncRNA SYISL recruits polycomb repressive complex 2 in C2C12 cells to suppress the expression of the fast-twitch fiber marker gene and myoblast differentiation (Jin et al., 2018). CircPTPN4 can function as a ceRNA to regulate nicotinamide phosphoribosyltransferase (*NAMPT*) expression by sponging miR-499-3p, thus participating in muscle fiber type switching in chicken primary myoblasts (Cai et al., 2022). The *longissimus dorsi* in cattle was shown to express CircMYBPC1 differently during adult and embryonic phases of development, and CircMYBPC1 interacted with the muscle gene MYHC by directly binding to miR-23a (Chen et al., 2021). Although many studies related to the formation of muscle fibers have been published, the detailed molecular mechanism remains unclear, especially in noncoding RNA and ceRNA in sheep.

The molecular process of the formation of muscle fibers in Tan sheep has not yet undergone a comprehensive examination. Therefore, clarifying the roles of the DEGs and noncoding RNAs can contribute to understanding the changes in the whole transcriptomes associated with the formation of muscle fibers in different sheep groups. In this study, the muscle fiber contents of *longissimus dorsi* and *biceps femoris* in Tan sheep and Dorper sheep were determined, and whole-transcriptome profiles in different groups (Tan sheep and Dorper sheep) and different tissues (*longissimus dorsi* and *biceps femoris*) of the same groups were obtained to compare the DEGs and noncoding RNAs. Finally, we built a thorough competing endogenous RNA (ceRNA) network to identify the genes that are most likely to be involved in the formation of muscle fibers. In-depth knowledge of the molecular processes underlying the development of muscle fibers in Chinese Tan sheep is provided in this report.

## Materials and methods

### Animal sample collection

Animal care and sample collection were authorized by the State Key Laboratory of Agricultural Biotechnology of the Agricultural University of China’s Animal Welfare Committee and carried out by the June 2004 amendments to the Regulations on the Control of Experimental Animals (Ministry of Science and Technology). Three unrelated male Tan sheep were used in the experimental group, while three unrelated Dorper sheep were used in the control group. Both groups of sheep were bred in Ningxia, China, under identical conditions until they were 8 months old, at which point they were killed on the same day. From these six sheep, samples of the *longissimus dorsi* and *biceps femoris* were taken and kept in liquid nitrogen or at −80°C until needed.

### Immunofluorescence staining experiment

To examine whether the proportion of slow-twitch fibers differed among different sheep groups, we performed immunofluorescence using a slow-twitch marker protein (MYH7 antibody). The fluorescein-labeled MYH7 antibody was used as a probe to detect the target antigen in the muscle tissue and to identify the location of the antigen. First, the frozen tissue of the *longissimus dorsi* and *biceps femoris* was sectioned. Slices of tissue were first fixed for 20 min at room temperature in 4% paraformaldehyde, followed by 20 min of penetration in 0.1% Triton X-100. Tissue sections were tagged overnight with an antibody (MYH7, 1/500) at 4°C following an hour of closure with 1% BSA. Tissue slices were washed three times with ×1 PBS the next day and then stained with Alexa Fluor 488-conjugated antibody (1/1,000) at room temperature for 1 h. Then, the tissue slices were washed with ×1 PBS, and 10 mg/ml Hoechst was added for 10 min.

### RNA extraction and sequencing

Following the manufacturer’s instructions, total RNA was extracted using TRIzol Reagent (Invitrogen, United States). RNA quality was evaluated using agarose gels at a 1% concentration. A K5500 spectrophotometer was used to measure the purity of the RNA (Kaiao, China). The Bioanalyzer 2100 system and the RNA Nano 6000 Assay Kit were used to measure RNA integrity and concentration (Agilent Technologies, United States). The mRNA/lncRNA/circRNA library was built using 5 µg of RNA from each sample. The HiSeq 4000 sequencing platform (Novogene, China) was used to build mRNA/lncRNA/circRNA sequencing libraries, and 150 bp paired-end reads were produced by the manufacturer’s instructions.

To create the small RNA library, 3 μg of the sample’s total RNA were employed. Using the NEBNext^®^ Multiplex Small RNA Library Prep Set for Illumina^®^ (NEB, United States) by the manufacturer’s instructions, sequencing libraries were built. Using DNA High Sensitivity Chips, the Agilent Bioanalyzer 2100 system assessed the sequencing library’s quality. The HiSeq 2500 sequencing platform (Novogene, China) was used to build small RNA sequencing libraries, and 50 bp single-end reads were produced. Fastq formatted raw data were initially processed using internal Perl programs.

### Bioinformatic analysis

The gene modeling annotation files and the reference genome (Oar v3.1) were accessed from Ensembl (https://asia.ensembl.org/index.html). Bowtie2 (LangmeadandS and Salzberg, 2012) was used to build the index of the reference genome, and HISAT2 (Pertea et al., 2016) was used to align paired-end clean reads to the reference genome. The mapped readings of each muscle sample were put together using StringTie (Pertea et al., 2016) with a reference-based method. Then, to identify lncRNAs, we assessed the assembled transcripts according to five criteria. The following transcripts were eliminated: 1) those with exon numbers <2, 2) those with lengths ≤200 bp, 3) those with known non-lncRNA annotations, 4) those with fragments per kilobase of exon per million fragments mapped (FPKM) < 0.5, and 5) Phylogenetic codon substitution frequency (PhyloCSF) (Sun et al., 2013). PFAM-scan (Kong et al., 2007), coding-noncoding-index (CNCI) (Finn et al., 2008), and coding potential calculator (CPC) (Kellis and Jungreis, 2011) were used to separate mRNAs from lncRNAs. All of the aforementioned tools predict that novel protein-coding transcriptome candidates are transcripts with coding potential, while novel lncRNAs are transcripts without coding potential. To determine the expression level of mRNA in each sample, FPKM (fragments per million fragments per exome) of coding genes was calculated using Cuffdiff (Trapnell et al., 2010). Differential expression analysis was performed using DESeq (Likun et al., 2010), with a default significant difference of two for fold change and a *q* value of 0.05.

Bowtie aligned small RNA tags to reference sequences to examine the expression and distribution of small RNA within the genome (LangmeadandS and Salzberg, 2012). Bedtools (https://bedtools.readthedocs.io/) was used to search for known miRNAs by matching them to entries in miRBase (http://www.mirbase.org/). MiRDeep2 (Friedlnder et al., 2012) was used to analyze the remaining reads to predict novel miRNAs. The following criteria were used to evaluate the expression levels of miRNAs and circRNA using TPM (Transcripts Per Kilobase of exon model per Million mapped reads) (Zhou et al., 2010). DESeq (Likun et al., 2010) was used to evaluate the differential expression, with a fold change threshold of 2 and a *p* value of 0.05.

CircRNAs were screened using find_circ (Memczak et al., 2013) and CIRI2 (Yuan and Zhao, 2015), and known and new circRNAs in each sample were calculated (read count) and normalized with TPM (Zhou et al., 2010). Based on the distance from the corresponding circRNA along the genome sequence, the closest protein-coding genes for circRNAs were found to mark circular RNAs. Using circBase ID, which refers to circBase annotation, all known circRNAs were given names, and novel circRNAs were given names following the rank number listed. Exonic circRNA, intronic circRNA, antisense circRNA, intergenic circRNA, 3′UTR circRNA, 5′UTR circRNA, and ncRNA circRNA were the seven categories used to categorize all circRNAs (Memczak et al., 2013). The expression levels of circRNAs were quantified by the number of junction-spanning reads, and an absolute *p* value < 0.05 and fold change ≥2 were considered to be significantly differentially expressed. The coding potential of circRNAs was predicted using the presence or absence of an internal ribosome entry site (IRES) independent of the 5′ cap structure on the circRNA. IRES finder (Zhao et al., 2018) was used to predict whether circRNA sequences have potential elements of IRES.

### Gene ontology and kyoto encyclopedia of genes and genomes enrichment analysis

DEGs, DE circRNA parental genes, DE lncRNAs, and DE miRNA target genes were subjected to functional and pathway enrichment analysis by Gene Ontology (GO) and Kyoto Encyclopedia of Genes and Genomes (KEGG) enrichment analysis. GOseq was based on the Wallenius noncentral hypergeometric distribution for GO enrichment analysis (Young et al., 2010). KOBAS v3.0 (Chen et al., 2011) was used to measure the statistical significance of enrichment in KEGG pathways.

### Prediction of lncRNAs, miRNAs, and CircRNAs and construction of CeRNA network

10 kb upstream and downstream of the coding genes were identified as potential regulatory targets of lncRNAs. Expression of lncRNAs used for trans-acting prediction was co-expressed with mRNA rather than correlated with mRNA location. Using Pearson’s correlation coefficients (r > 0.90 or r < −0.90) as a classifier, the expressed correlation between non-coding genes and genes was computed. Using miRanda (Enright et al., 2003), PITA (Marc et al., 2004), and RNAhybrid (Ventsislav et al., 2005), the prediction of miRNA target genes was carried out. The following threshold settings for PITA were established: maximum target length is 50,000; energy cut-off is 10, and 0.05 is the cut-off for the *p*-value. The following parameters were entered for RNAhybrid: utr: 3utr.fa; mir: mature. fa. To ensure the accuracy of our findings, we set the Smith-Waterman hybridization alignment match score higher than 140 and the minimum free energy of the duplex structure lower than −10. We created lncRNA/circRNA-miRNA‒mRNA networks with lncRNA/circRNA acting as decoys, miRNA acting as the core, and mRNA acting as the target. Using Cytoscape (Michaele et al., 2011), the lncRNA, circRNA, miRNA, and mRNA interactions were created and visualized.

### Validation of differentially expressed transcripts

RT-qPCR validated the expression pattern of randomly selected differentially expressed transcripts from the longissimus dorsi and biceps femoris. FastKing gDNA Dispelling RT SuperMix and SuperReal PreMix Plus (Tiangen, China) mRNA detection assays were used for miRNA tests. The miRcute Plus miRNA First-strand cDNA Kit and miRcute Plus miRNA qPCR Kit (Tiangen, China) were used for microRNA tests. RT primers and a bespoke qRT‒PCR quantitative kit (GenePharma, China) were used for circRNA detection. The RNase R- and control groups (without RNase R) were produced and translated into cDNA, and each circRNA was amplified using the primers indicated to visualize the back-splicing junction of the circRNA (Myriam and Gorospe, 2018; Heng et al., 2019). The back-splicing sites were then detected by sequencing the products. GAPDH and U6 small nuclear RNA genes were chosen as endogenous control genes (all primers are shown in Supplementary Table S12). Three biological replicates and triple reactions for each sample were used for all qPCR validations. Following amplification, the products were validated by agarose gel electrophoresis and Sanger sequencing, and the 2^−ΔΔCt^ method was used to determine the relative transcript abundance.

### Cell culture and vector construction

Primary sheep myoblasts were isolated and cultured from embryo sheep leg muscle. Myoblasts and HEK293T cells (ATCC, United States) were cultured in high-glucose DMEM supplemented with fetal bovine serum (Hyclone, United States) and double antibiotics (1% penicillin and streptomycin). To induce myoblasts differentiation, cells were switched to a differentiation medium (DMEM; 2% horse serum) in nearly 90% confluence. Small interfering RNA (siRNA) against sheep circ_0017336 were designed and synthesized by Genepharma (Suzhou, China), and a nonspecific duplex was used as the control. Sheep myoblasts were transfected with 100 nM siRNA using Lipofectamine 2000. The si-circ_0017336 sequences are 5′-UUU​GGA​GAU​AGC​AGG​GCU​GTT-3′, 3′CAG​CCC​UGC​UAU​CUC​CAA​ATT-5′. The fragment of the FBXL5 3′ UTR, including the binding site of miR-23a, was amplified and inserted into the psicheck2 vector (Promega, United States) at the 3′ end of the Renilla gene using restriction enzymes XhoI and NotI and T4 DNA ligase (TaKaRa, China). Psicheck2-FBXL5-M and psicheck2-FBXL5-W constructs were verified by sequencing.

### Dual-luciferase reporter analysis

When the cell confluence reached about 80%, the miR-23a mimics and psicheck2-FBXL5-W or psicheck2-FBXL5-M were co-transfected into HEK293T cells using Lipofectamine 2000. After incubation for 24 h, Dual-luciferase activity was measured using an automatic microplate reader (Molecular Devices, United States), and the Renilla luciferase activity was normalized against firefly luciferase activity. The miR-23a mimics sequences are 5′-AUC​ACA​UUG​CCA​GGG​AUU​UCC​A-3′, 3′-GAA​AUC​CCU​GGC​AAU​GUG​AUU​U-5′.

### Analytical statistics

The means and standard error of the means are used to express data (SEM). Levene’s test was used to check for homogeneity of variances, and then Student’s *t*-test was used to determine significance. At *p* < 0.05, differences were deemed statistically significant.

## Results

### The proportion of slow muscle fibers in the *Biceps femoris* and *Longissimus dorsi* in sheep

**FIGURE 1:**
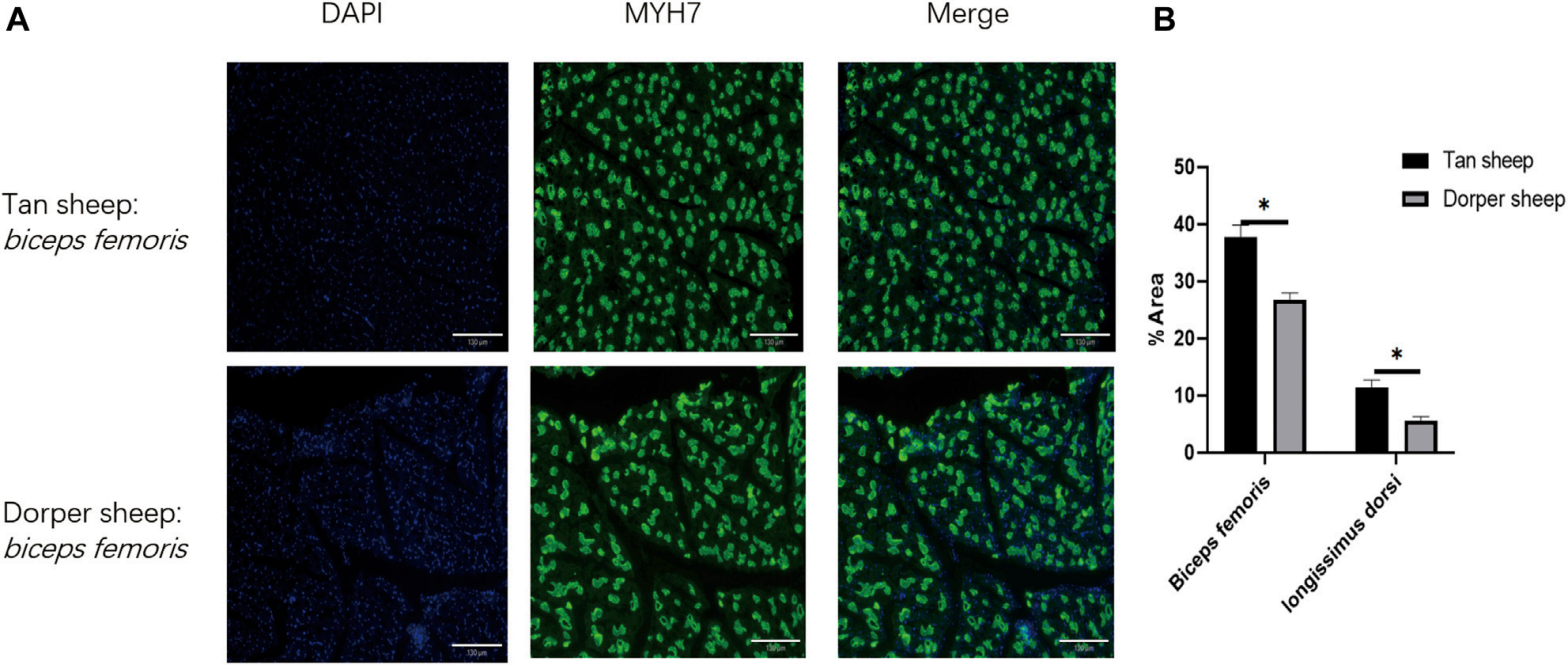
The difference of the slow-twitch fiber ratio of longissimus dorsi and Biceps femoris muscle between Tan sheep and Dorper sheep. **(A)** Frozen sections were immunofluorescently stained with MyH 17 antibody. “DAPI” is a staining solution suitable for the nuclear staining of common cells and tissues. “Merge” is to fuse the stained nucleus with the target protein. **(B)** Statistical chart of immunofluorescence staining results.

According to the results of immunofluorescence staining, the proportions of slow-twitch fibers in the *longissimus dorsi* and *biceps femoris* of Tan sheep were 11.4% and 37.7%, respectively, and its proportions were 5.6% and 26.8% in the *longissimus dorsi* and *biceps femoris* of Dorper sheep. Tan sheep had considerably more slow-twitch muscle fibers than Dorper sheep did (*p* < 0.01) in their *longissimus dorsi* and *biceps femoris* (Figures 1A,B).

### Blast analysis of transcriptome sequencing

**FIGURE 2:**
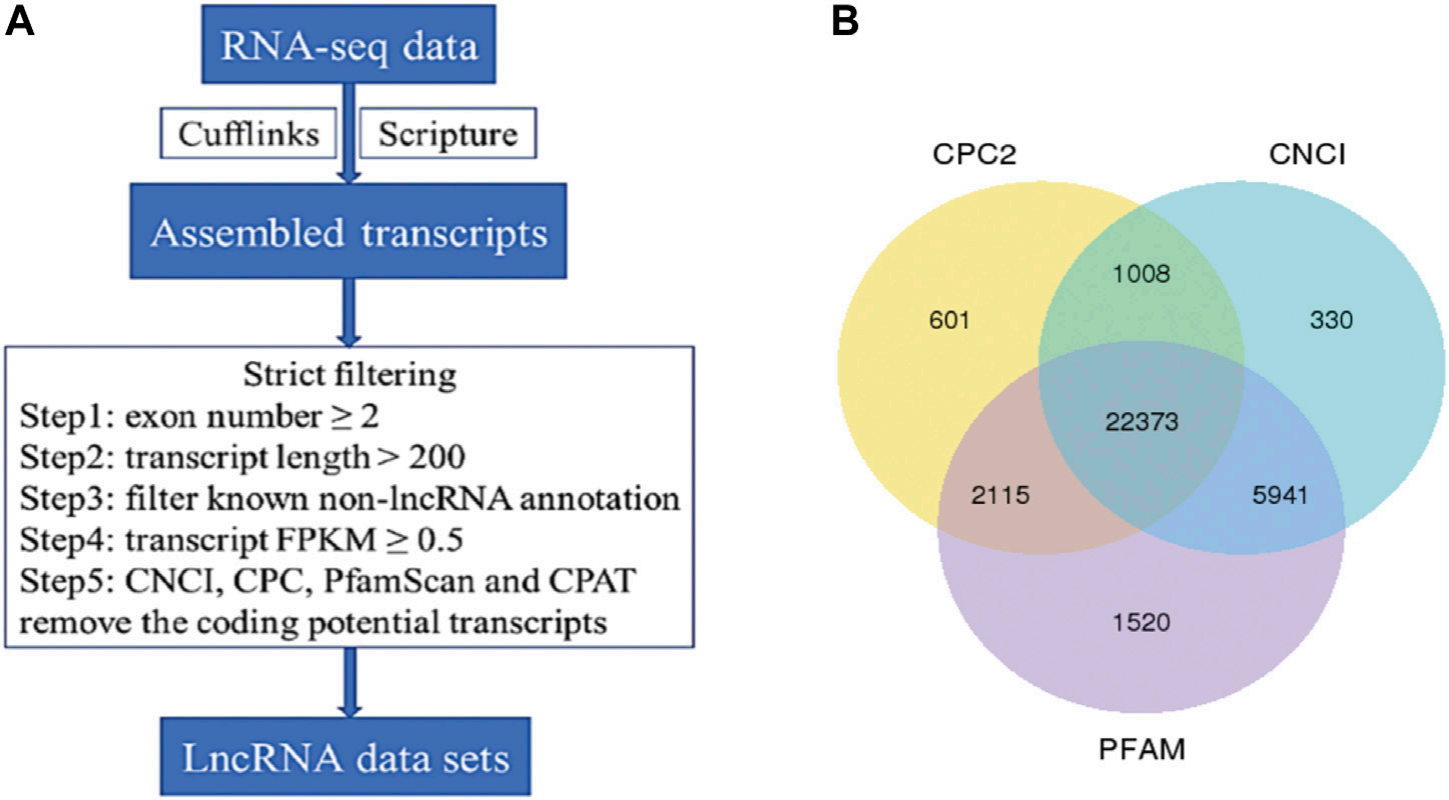
Identification of long non-coding RNAs (IncRNA) in muscle transcriptome. **(A)** Workflow for IncRNA identification. **(B)** Candidate IncRNAs were identified by using three applications: CNCI, coding-non-coding-index; CPC, coding potential calculator; PFAM-scan which detect and remove putative protein-coding transcripts.

**FIGURE 3:**
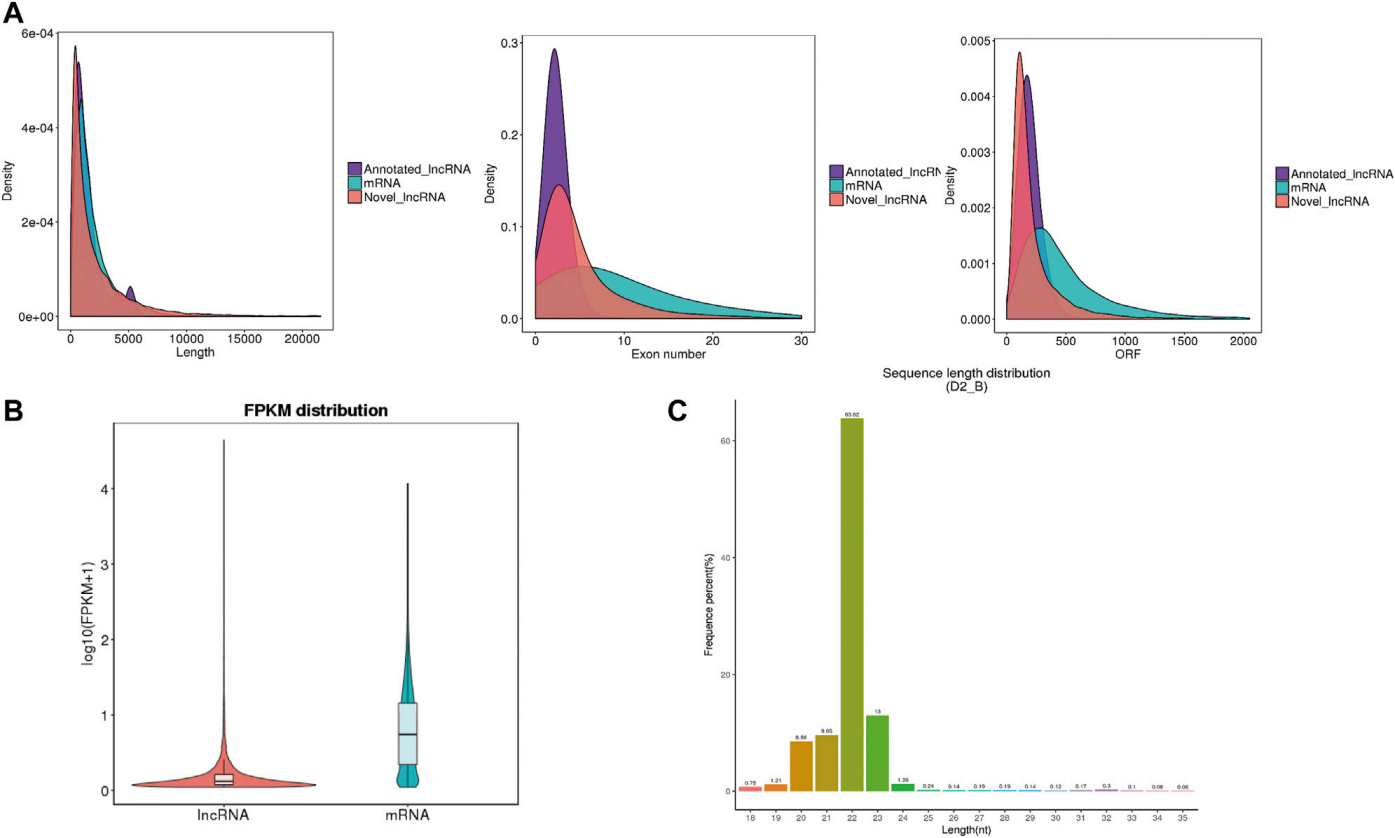
Genomic architecture and expression level of IncRNAs and miRNAs. **(A)** Comparison of mRNA transcript length, exon number and open reading frame (ORF) length. **(B)** Expression level of IncRNAs and mRNAs, calculated as log10(FPKM+1). **(C)** Length distribution of all identified small RNAS.

The whole-transcriptome profiling of *longissimus dorsi* and *biceps femoris* tissues from Dorper sheep and Tan sheep were obtained to evaluate the genes involved in the formation of muscle fibers. The 12 samples used for the RNA sequencing libraries yielded an average of 95.74 million clean reads, and 83.55% to 90.07% of these reads were specifically aligned to the reference genome Oar v3.1. At least 85% of the readings were equal to or greater than Q30 (Supplementary Table S1-1). A total of 22,823 mRNAs, 224 known lncRNAs, and 22,373 unique lncRNAs were found in the muscle samples after additional filtering (Figure 2A) and removal of possible coding transcripts that were identified using CNCI, CPC, and PFAM (Figure 2B). The gene structure, expression, and sequence conservation of lncRNAs and protein-coding genes were compared. As lncRNA genomic characterizations were compared to those of mRNAs, it was discovered that the length range of their transcripts was similar; more lncRNAs had 2–4 exons than mRNAs, and they also had shorter open reading frames (ORFs) and lower FPKM values (Figures 3A,B).

With at least two independent reads crossing the back splice junction, a total of 3651 circRNAs were found. Exons, introns, and intergenic regions all serve as various circRNA source locations. Exons were more likely to produce circRNAs in this study; 3141 circRNAs were produced from exons of 1810 genes, whereas 197 circRNAs were produced from intronic regions of 175 genes, and our data indicated that the remaining circRNAs were intergenic. CircRNAs ranged in length from 192 nucleotides to 93,278 nucleotides and were mainly located on chromosome 1 (Supplementary Table S2). Interestingly, 4611 internal ribosome entry site (IRES) were predicted for circRNAs and that the highest score may reach 0.997 (Supplementary Table S12).

Additionally, for the small RNA-Seq libraries, rigorous filtering yielded an average of 16.02 million clean reads, with approximately 95% of these clean reads matched to the sheep reference sequence (Supplementary Table S1-2). Most clean reads ranged from 20 to 24 nt (Figure 3C). There were 238 known miRNAs in each sample, according to statistical statistics (Supplementary Table S2). A total of 141 mature miRNAs were annotated, and 97 new mature miRNAs were found (Supplementary Table S2).

### Differential expression of genes and noncoding RNAs (LncRNAs, miRNAs, and circRNAs) between Tan sheep and Dorper sheep

**FIGURE 4:**
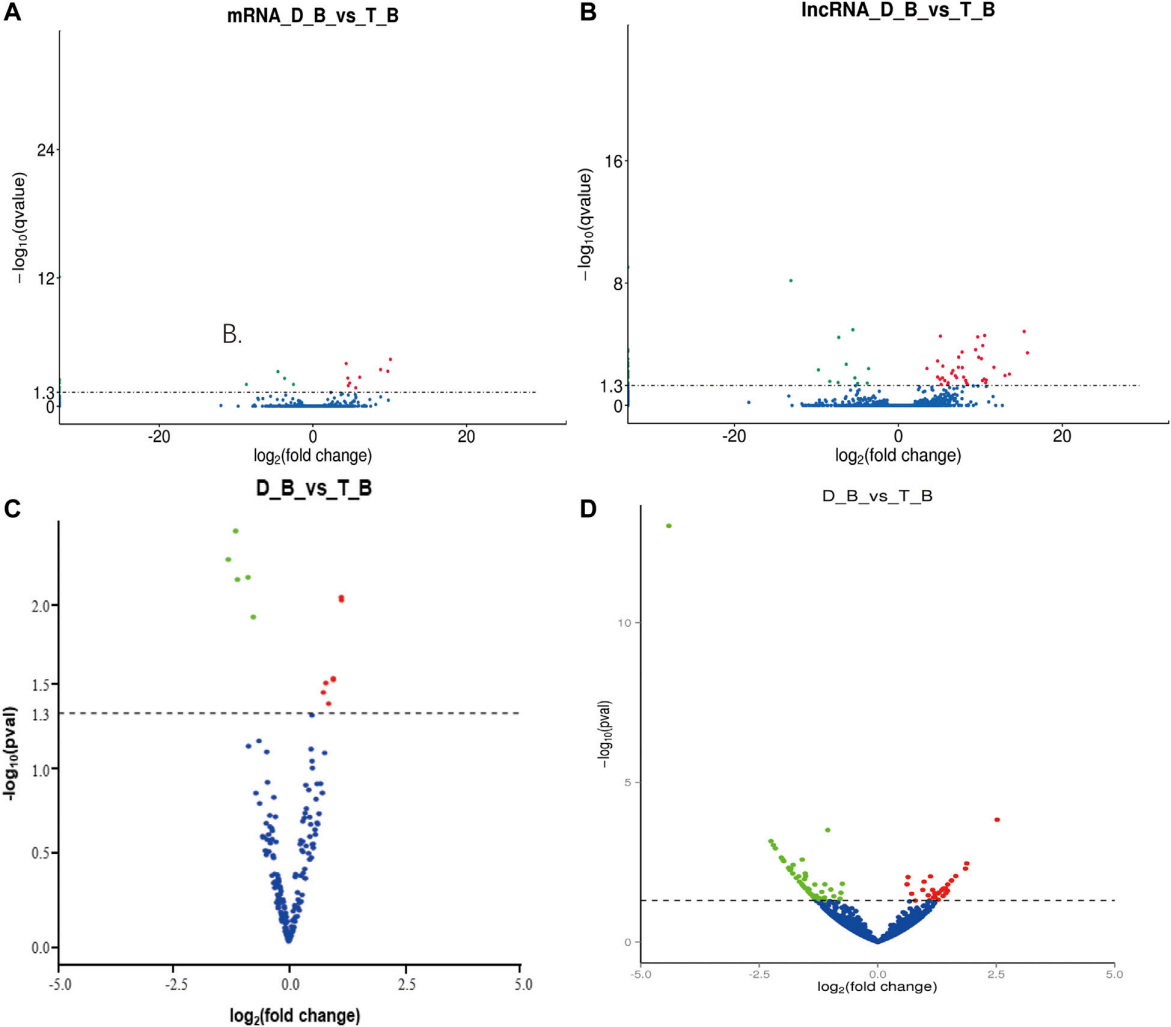
Differentially expressed mRNAs and non-coding RNAs in Tan sheep and Dorper sheep. **(A)** Expression profiles of mRNA in biceps femoris of Tan sheep and Dorper sheep. **(B)** Expression profiles of IncRNA in biceps femoris of Tan sheep and Dorper sheep. **(C)** Expression profiles of miRNA in biceps femoris of Tan sheep and Dorper sheep. **(D)** Expression profiles of circRNA in biceps femoris of Tan sheep and Dorper sheep. In the volcano plots, green points represent downregulated RNAs, while red points represent upregulated RNAS. “D_B” is the biceps femoris group of the Dorper sheep. “T_B” is the biceps femoris group of the Tan sheep.

In *biceps femoris* tissues, twelve DEGs were obtained between Tan sheep and Dorper sheep, where 11 were upregulated and 24 were downregulated (Supplementary Table S3; Figure 4A). Several DEGs were specifically expressed in Tan sheep, such as SFSWAP, or Dorper sheep, such as ASAH1. These genes may regulate the formation of muscle fibers.

Similarly, 172 DELs were discovered in the *biceps femoris* between Tan and Dorper sheep, of which 28 were upregulated and 144 were downregulated (Supplementary Table S5; Figure 4B). Several DE lncRNAs were particularly expressed in Tan sheep, including LNC 000295, LNC 000300, and LNC 000306, or Dorper sheep, including LNC_017854, LNC_02118,6, and LNC_015846. In the *biceps femoris*, there was a total of 15 differential expression miRNAs, of which 5 were upregulated and 7 were downregulated between Tan sheep and Dorper sheep (Supplementary Table S8; Figure 4C). MiR-133 (Martin et al., 2015), miR-370 (Zhang et al., 2021), and miR-148 (Yin et al., 2020) have been revealed to be important in the development of muscle fibers among these differentially expressed miRNAs. Additionally, 95 DECs were discovered in the *biceps femoris* between Tan sheep and Dorper sheep, of which 59 were upregulated and 36 were downregulated (Supplementary Table S10; Figure 4D).

In *longissimus dorsi* tissues, twenty-five DEGs were found between Tan sheep and Dorper sheep, including 12 upregulated and 13 downregulated in Tan sheep, which have more than 2-fold differential expression (Supplementary Table S3). These DEGs included F-box and leucine-rich repeat protein 5 (*FBXL5*) and myosin light chain 3 (*MYL3*), which are related to the regulation of the formation of muscle fibers. Several DEGs were specifically expressed in Tan sheep, such as FBXL5, or Dorper sheep, such as SFSWAP.

In all, 214 DELs were obtained in the *longissimus dorsi* tissues of Tan sheep and Dorper sheep, of which Tan sheep had 101 upregulated and 113 downregulated DELs (Supplementary Table S5). Several DE lncRNAs were specifically expressed in Tan sheep, such as LNC_003872, LNC_01870,8, and LNC_021269, or Dorper sheep, such as LNC_008982, LNC_00889,6, and LNC_001693. These lncRNAs may control how muscle fibers are formed. Three known miRNAs were expressed overall at lower levels in Tan sheep than in Dorper sheep, whereas one known miRNA was expressed at higher levels (Supplementary Table S8). One of these miRNAs with variable expression, miR-23a, has been linked to the development of muscle fibers (Chen et al., 2021). In the *longissimus dorsi* tissues of Tan sheep and Dorper sheep, a total of 91 DECs were also discovered, of which 45 were upregulated and 46 were downregulated (Supplementary Table S10).

### Functional analysis of differentially expressed transcripts

**FIGURE 5:**
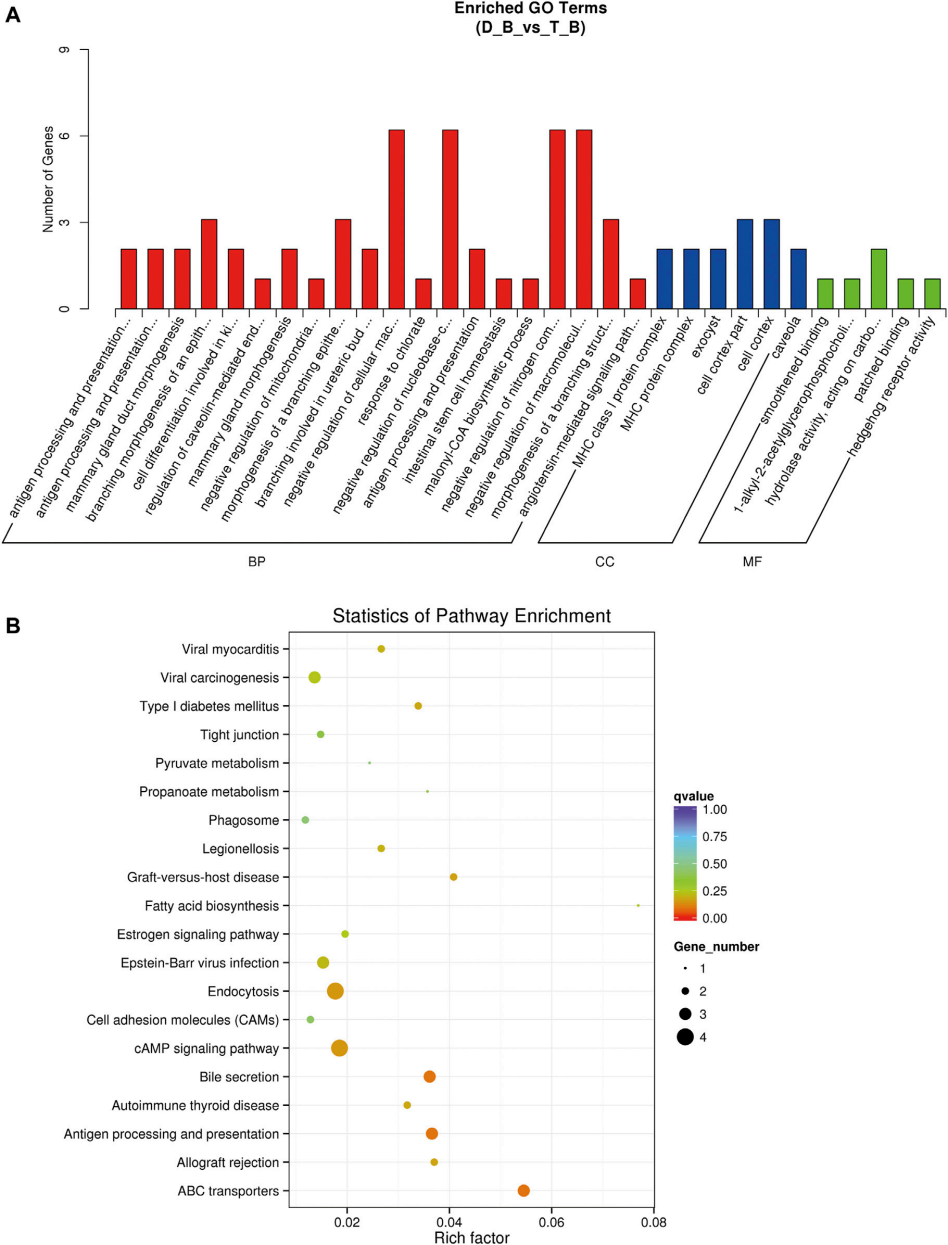
GO and KEGG enrichment analysis in biceps femoris of Tan sheep and Dorper sheep. **(A)** GO enrichment annotation of mRNAs. **(B)** KEGG annotation of mRNAs. “D_B”is the biceps femoris group of the Dorper sheep. “T_B” is the biceps femoris group of the Tan sheep.

GO and KEGG pathway analysis were carried out to assess the potential roles of differentially expressed genes, lncRNAs, miRNAs, and circRNAs. Three functional categories were carried out of the GO pathways for DEGs (biological process, cellular component, and molecular function). The biological processes involved in the development of muscle fibers, such as calcium ion transport (GO: 0006816), oxidation-reduction process (GO: 0055114), muscular system process (GO: 0003012), and regulation of myoblast differentiation (GO: 0045661), were also significantly enriched. The CAMP signaling pathway and metabolic pathways were significantly enriched according to KEGG enrichment analysis (Supplementary Table S4; Figures 5A,B). The development of muscle fibers also includes several important metabolic and myoblast differentiation-related genes, including ATP6V0A1 and MYL3.

Two separate algorithms—cis (genomic location) and trans (expression correlation)—were used to predict the target genes of the identified lncRNAs to elucidate their potential roles in the formation of muscle fibers, 2,154 cis-acting lncRNAs target a total of 720 genes among them, while 446 trans-acting lncRNAs have a total of 163 target genes. The GO pathway of cis-lncRNAs was highly enriched in negative regulation of glycolysis (GO: 0006110), muscle fiber development (GO: 0048741), and ATPase activity (GO: 0042623), and skeletal muscle contraction (GO: 0003009) (Supplementary Table S6). KEGG enrichment was significantly enriched in the metabolic pathway muscle atrophy; GO annotation of trans-lncRNAs was significantly enriched in positive regulation of skeletal muscle tissue development (GO: 0048643) and muscle cell differentiation (GO: 0042692); KEGG enrichment was significantly enriched in glycolysis, TCA cycle, and Wnt signaling pathway (Supplementary Table S7). GO annotation and KEGG enrichment analysis of the targets showed miRNAs were mainly enriched in metabolic pathways (Supplementary Table S9). The parental genes of DE circRNAs were largely enriched in myofibrils (GO: 0030016) and glycogen metabolic pathways (GO: 0005977), according to GO analysis. DE circRNAs were primarily enriched in the Ca^2+^ signaling pathways, FoxO signaling pathways, and AMPK signaling pathways, according to KEGG pathway analysis (Supplementary Table S11).

### Validation of differentially expressed transcript expression

**FIGURE 6:**
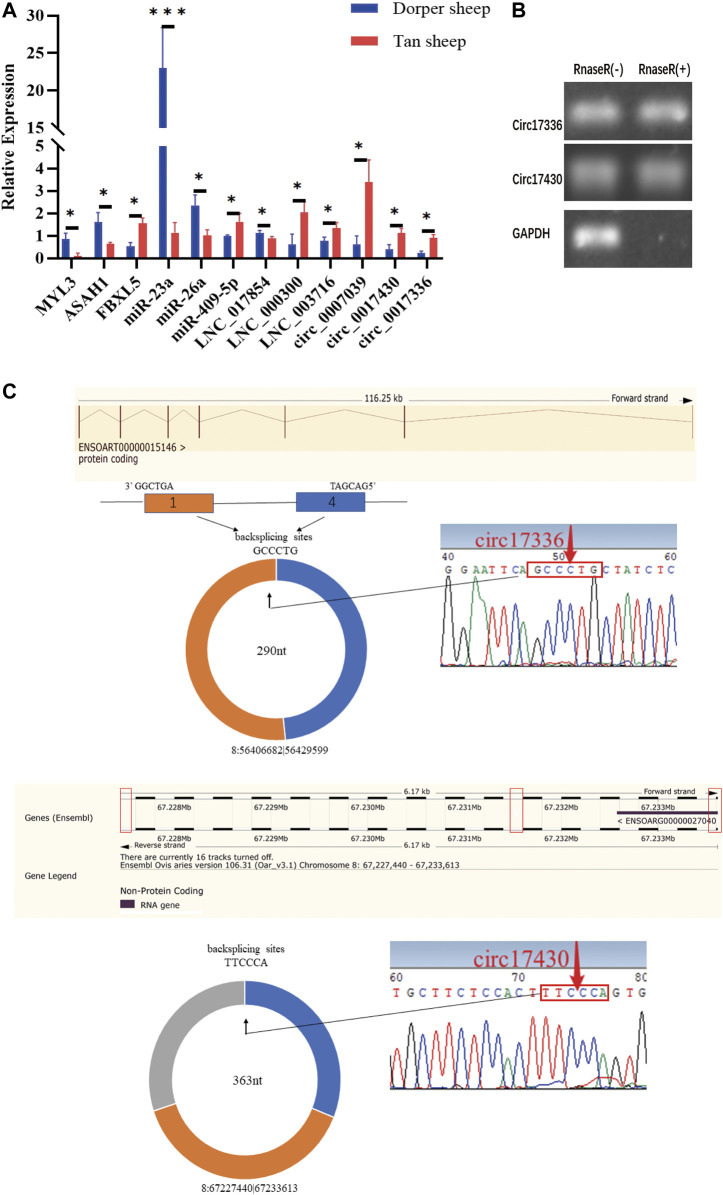
**(A)** The expression levels of mRNA, miRNA, IncRNA and circRNA were verified by qPCR. **(B)** The results obtained from use of Rnase R indicate that the expression levels of circ_0017336 and circ_0017430 are resistant to Rnase R. “Rnase R (+)” indicates RNA treated with RNase R: “Rnase R (−)” indicates untreated RNA. **(C)** The backsplice junctions of circ_0017336 and circ_0017430 were verified with Sanger sequencing. The red arrows indicate head-to-tail back-splicing sites of circRNAs.

To validate the RNA-seq results, *MYL3*, *ASAH1*, miR-409-5p, circ_0017336, circ_0007039, LNC_0017854, LNC_003716, and LNC_000300 were selected, and their expression patterns in *biceps femoris* tissue of Tan sheep and Dorper sheep were examined using qPCR. *FBXL5*, miR-23a, miR-26a, and circ_0017430 were also selected, and their expression patterns in *the longissimus dorsi* of Tan sheep and Dorper sheep were examined using qPCR (Figure 6A). RT–qPCR verified that the expression patterns of randomly above differentially expressed transcripts were highly consistent with those obtained by RNA sequencing. Notably, RNase R digestion assays further demonstrated that circ_0017336 and circ_0017430 had a circular structure (Figure 6B), and the back splice junctions of circRNAs were confirmed before validation (Figure 6C).

### Construction of CeRNA regulatory network

**FIGURE 7:**
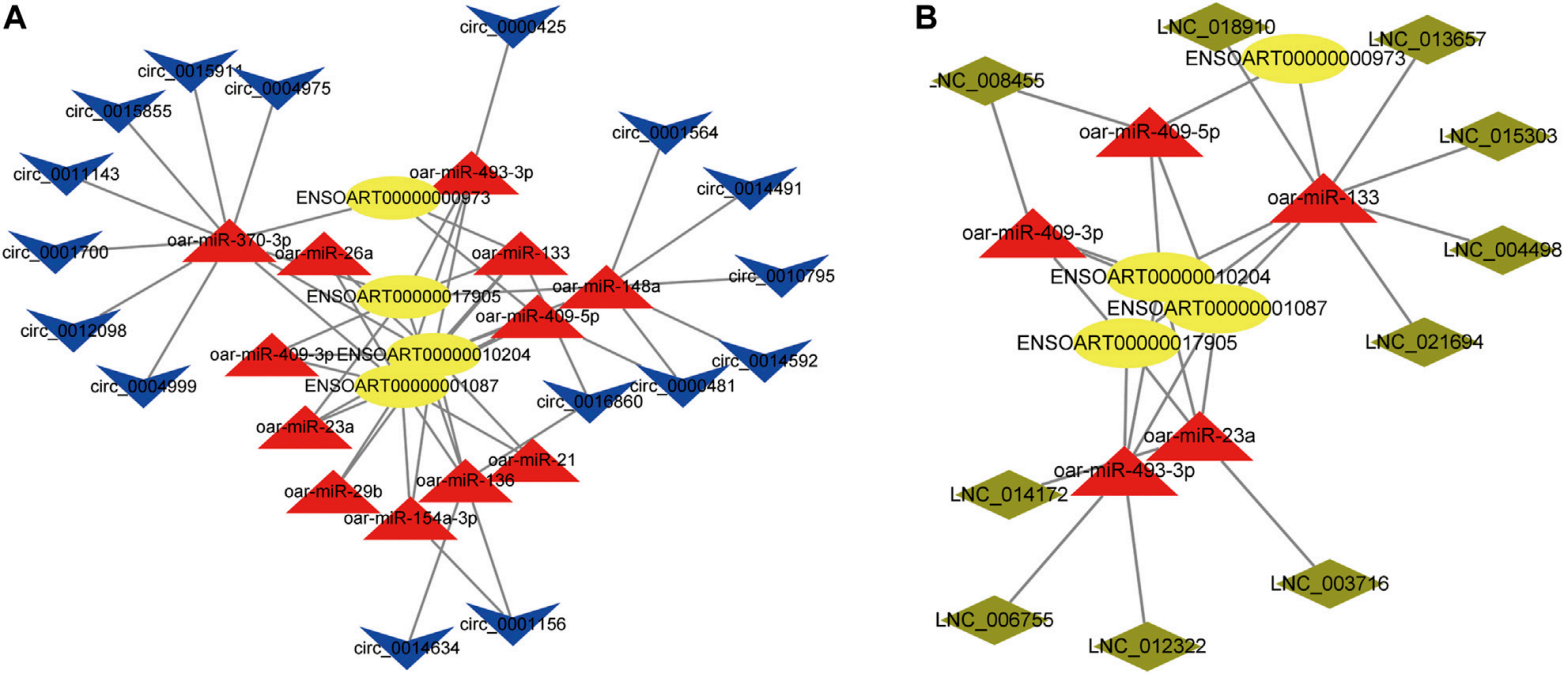
**(A)** CircRNA-miRNA-mRNA interaction network specifically expressed in Tan sheep. **(B)** LncRNA-miRNA-mRNA interaction network specifically expressed in Tan sheep. The ovals, triangles, parallelograms and V types represent mRNAs, miRNAs, IncRNAs and circRNAs, respectively.

We found lncRNA and circRNA gene pairs with miRNA binding sites based on the ceRNA hypothesis, and we constructed lncRNA‒miRNA-gene pairs and circRNA-miRNA-genes with lncRNA and circRNA acting as decoys, miRNA as the core, and mRNA as the target. The ceRNA network generated above was then visualized with Cytoscape software, using various shapes to represent various types of RNAs. The specific circRNA-miRNA-gene networks and lncRNA‒miRNA-gene networks closely related to the formation of muscle fibers in Tan sheep are shown in Figures 7A,B and a summary table listing all possible functioning ceRNA networks was added to Supplementary Table S14. Furthermore, from the ceRNA network, we observed some ceRNA subnetworks, which showed that circ_0017336 and its target FBXL5, ACACB and EXOC6 “talked” to each other through the same miR-23a response elements, whereas LNC_014172 and LNC_003716 “talked” to their targets through miR-23a response elements, respectively. Therefore, we speculate that these subnetworks may play a key role in the formation of muscle fiber.

**FIGURE 8:**
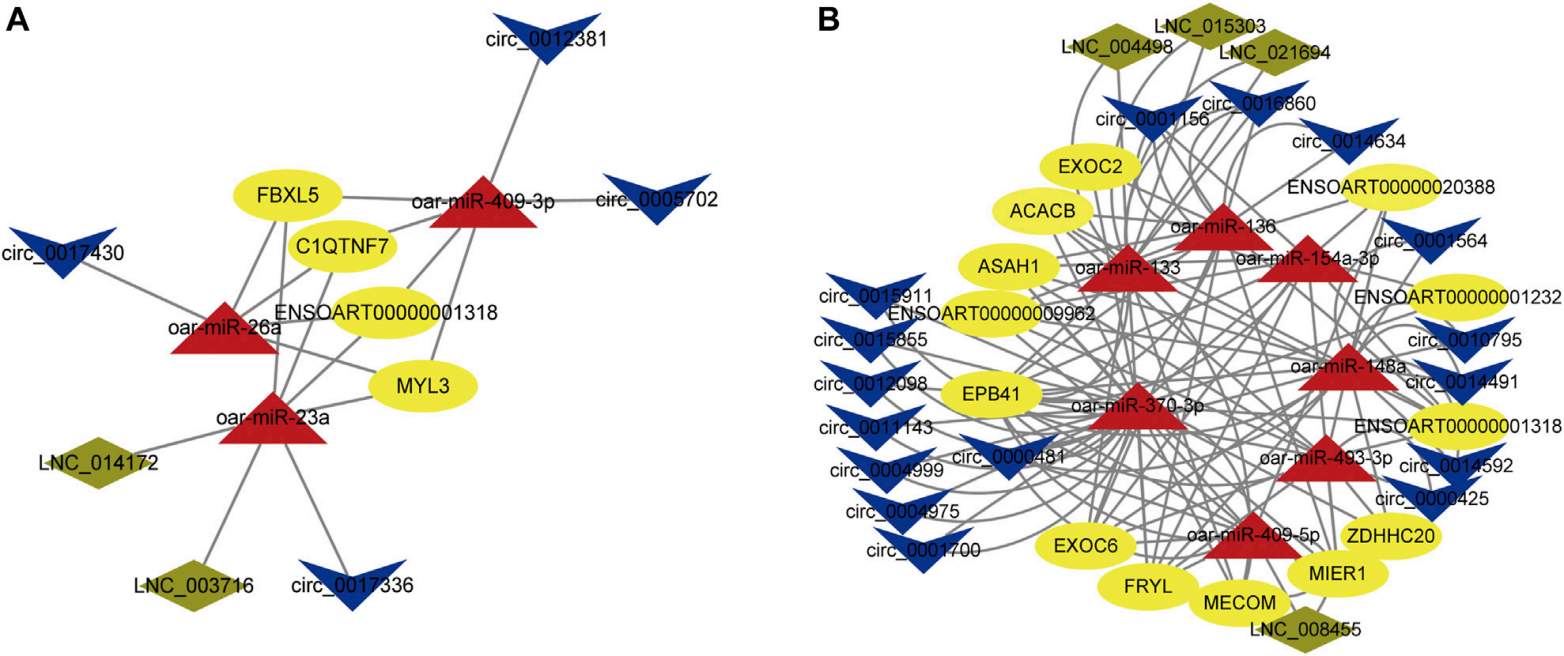
ceRNA network construction in differnt muscle tissues. **(A)** LncRNA/circRNA-miRNA-mRNA interaction network was constructed based on different expression levels in the longissimus dorsi of Tan sheep and Dorper sheep. **(B)** LncRNA/circRNA-miRNA-mRNA interaction network was constructed based on different expression levels in the biceps femoris of Tan sheep and Dorper sheep. The ovals, triangles, parallelograms and V types represent mRNAs, miRNAs, IncRNAs and circRNAs respectively.

In addition, we also constructed ceRNA networks in the *longissimus dorsi* and *biceps femoris* tissues. In the *longissimus dorsi*, the network shows possible interactions among 2 DE lncRNAs, 3 DE miRNAs, 4 DE circRNAs, and 4 DE mRNAs (Figure 8A; Supplementary Table S14). For example, circ_0017336 were identified as ceRNA of miR-23a, which targeted MYL3, FBXL5, and C1QTNF7; LNC_003716 and LNC_014172 regulated ENSOART00000001318 by competing miRNA response elements of miR-23a. The network depicts potential connections among 4 DE lncRNAs, 7 DE miRNAs, 16 DE circRNAs, and 13 DE mRNAs in the *biceps femoris* (Figure 8B; Supplementary Table S14). For instance, ASAH1 and circ_0000481 potentially create a ceRNA network through miR-409-5p as a bridge. These findings imply that genes and noncoding genes that exhibit differential expression in Tan sheep and Dorper sheep might work together to control the formation of muscle fibers *via* an interaction network, and their regulatory patterns are also different in different tissue sites.

### miR-23a directly bound FBXL5

**FIGURE 9:**
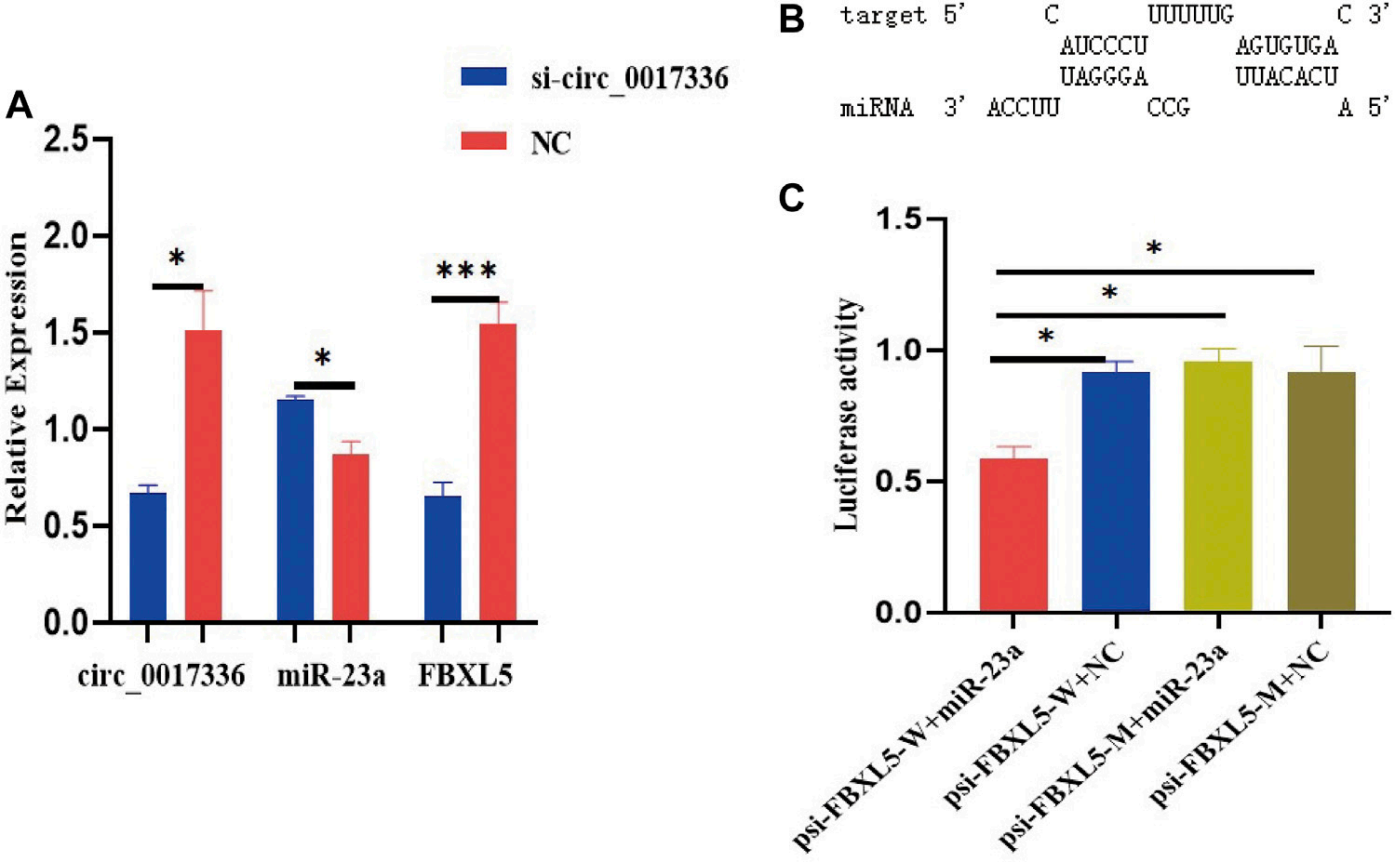
**(A)** si-circ_0017336 promotes the expression of miR-23a and inhibits the exprsion of FBXLS in sheep myoblasts. **(B)**. The binding sites of miR-23a and FBXLS were predicted by RNAhybrid software. **(C)** Dual-luciferase reporter gene experiments verified that miR-23a directly targets FBXL5. indicates *p* < 0.05, ***indicates *p* < 0.001, NC is the control group.

To investigate whether circ_0017336 acts as a competing endogenous RNA in sheep myoblasts, the interfering fragment si-circ_0017336 was transfected into sheep myoblasts to significantly inhibit the expression level of circ_0017336. The changes of miR-23a and FBXL5 expression level were detected after 3 days of differentiation of myoblasts treated with si-circ_0017336, and it was found that the interference of circ_0017336 significantly increased the expression of miR-23a in skeletal muscle cells, but suppressed the expression of FBXL5 (Figure 9A). To further validate the targeting relationship between miR-23a and FBXL5, the prediction software RNAhybrid revealed that FBXL5 had putative miR-23a binding sites (Figure 9B). To verify whether miR-23a directly targets FBXL5, miR-23a mimics were co-transfected with psi-CHECK2 dual-luciferase reporters containing the 3′UTR of FBXL5 into 293T cells for luciferase activity analysis, and we found that overexpression of miR-23a reduced the luciferase activity (Figure 9C). These results revealed that FBXL5 could indeed bind miR-23a directly.

## Discussion

The formation of muscle fibers is a polygenic trait in sheep, mainly determined by genetic factors, and is an important determinant of meat quality characteristics. Previous studies have revealed a closely association of the type of muscle fiber with meat quality traits due to the effects on the postmortem metabolic rate in the conversion of muscle to meat. The association between more slow-twitch fibers and better meat quality has been observed in a variety of species, including sheep (Valin et al., 1982), pigs (Bowker et al., 2005; Zhou et al., 2021), chickens (Ouyang et al., 2017), and calves (Maltin et al., 1998). Two mechanisms have been suggested for the muscle fiber pattern in Tan sheep. According to the research mentioned above, one theory proposes that the difference in the amount of slow-twitch fiber, with Tan sheep having more and Dorper sheep having less, is what causes the phenotypic. An alternative explanation claims that the difference is the result of an epigenetic regulatory process, possibly involving noncoding RNAs.

Given that some essential genes linked to specific phenotypes or significant biological processes have been identified using high-throughput RNA sequencing, it may be possible to understand the mechanisms underlying the formation of muscle fiber patterns. Numerous muscle tissue transcription profiles in animals have been studied using RNA sequencing, but similar studies have not been conducted in Tan sheep. To determine the critical elements involved in the formation of muscle fibers, we evaluated, for the first time, the differences in the expression profiles of mRNAs, lncRNAs, and circRNAs in the *longissimus dorsi* and *biceps femoris* tissues of two sheep breeds. Additionally, we found that a more complex ceRNA regulatory network is formed in the *longissimus dorsi* than in the *biceps femoris*. As studies have shown that slow-twitch fibers have higher mitochondrial content and oxidative phosphorylation capacity, and the ratio of slow-twitch fibers in the *longissimus dorsi muscle* is higher than that in the *biceps femoris* muscle, which makes it possible that the *longissimus dorsi* muscle has more glycolysis, oxidative phosphorylation, ad some metabolism-related genes to form a more complex ceRNA regulatory network.

Different metabolic and contractile characteristics exist between slow-twitch and fast-twitch fibers. Fast-twitch fibers have low oxidative metabolism and low mitochondrial content, while slow-twitch fibers have a high oxidative capability and large mitochondrial content. FBXL5, a member of the F-box protein family, regulates iron homeostasis in cells and systems by promoting the degradation of iron regulatory protein 2, and is an essential sensor for bioavailable iron (Ruiz and Bruick, 2014; Moroishi et al., 2014). The lack of iron in the body is closely related to the quality and function of skeletal muscle (Scherbakov et al., 2019), and we think that the formation of muscle fibers may be affected by the regulation of iron content by FBXL5. The acetyl-CoA carboxylase beta (ACACB) and ATPase H+ transporting V0 subunit a1 (ATP6V0A1) genes were significantly upregulated in Tan sheep, both of which were enriched in the oxidative phosphorylation pathway and consistent with the fact that Chinese native sheep have a better flavor than sheep from other regions. The main function of EFHB is to bind calcium ions (Rosado, 2021), triggering a Ca^2+^-dependent transport pathway (Wu et al., 2002; Chin, 2005), causing the formation of skeletal muscle fibers. Additionally, MYL3 showed differential expression in the muscle tissues of Tan sheep and Dorper sheep, which was consistent with earlier research that also deemed these genes to be key candidates for muscle growth and development (Ouyang et al., 2017). According to GO analysis, MYL3 is mostly involved in muscle contraction. Additionally, the specific roles of SFSWAP and two novel transcripts (ENSOART00000001243 and ENSOART00000001318) are unknown and are differentially expressed in both tissues. While ENSOART00000001243 and ENSOART00000001318 are downregulated in both *longissimus dorsi* and *biceps femoris* tissues, SFSWAP was interestingly increased in *longissimus dorsi* tissues but downregulated in *biceps femoris* tissues. The varied functions of these genes during the formation of these two tissues may be related to these consistent or inconsistent trend changes in expression trends, which are worthy of future analysis.

Noncoding RNAs act as epigenetic regulators of the expression of protein-coding genes in eukaryotes, which can control the expression at both transcriptional and post-transcriptional stages (Kornienko et al., 2013), and the large-scale ceRNA networks composed of noncoding RNAs and mRNA are important for regulation in various physiological and pathological processes (Salmena et al., 2011; Ala et al., 2013). Numerous miRNAs are important regulators of the growth of muscle fibers. For instance, miR-23a affects the expression of the MYHC gene and the development of muscle cells (Wang et al., 2012; Chen et al., 2021). The constructed ceRNA network results also demonstrated that DE circRNAs competed with miR-23a for binding and may function as ceRNAs to regulate the levels of ACACB, FBXL5, and EXOC6, respectively. This confirms the significance of miR-23a not only in its functions but also as a bridge in the ceRNA mechanism for the formation of muscle fibers. We hypothesized that several subnetworks within the network, such as circ_0017336-miR-23a-FBXL5, may be extremely important in controlling the formation of muscle fibers. RT-qPCR results showed that there was a ceRNA relationship between circ_0017336 and miR-23a as well as FBXL5 during sheep myogenic differentiation, and the dual luciferase reporter systems further verified that miR-23a directly targets FBXL5 for regulation. However, their roles and relationships still require confirmation. Our knowledge of the formation of muscle fiber phenotypes in sheep is expected to progress with the identification of new functions for lncRNAs and circRNAs.

Taken together, our data provide evidence for interactions that have high functional specificity in the formation of muscle fibers and that are consistent with the ceRNA hypothesis. This study provides new insights into the complex molecular mechanisms underpinning sheep meat traits variation but understanding exactly how the different type of muscle fiber are formed in the Tan sheep and Dorper sheep will require additional experiments.

## Conclusion

The current study paints a thorough picture of the differences between Tan sheep and Dorper sheep in the whole-transcriptome profiles of the *longissimus dorsi* and *biceps femoris* tissues. Tan sheep had much more slow-twitch fibers than Dorper sheep had in their *longissimus dorsi* and *biceps femoris*. Among two sheep breeds, 60 DEGs were discovered, including ACACB, ATP6V0A1, ASAH1, EFHB, MYL3, C1QTNF7, SFSWAP, and FBXL5. Act according to our research, numerous lncRNAs, miRNAs, and circRNAs play a crucial role in crucial biological processes related to the development of muscle fiber pathways, such as the FoxO signaling pathway, the AMPK signaling pathway, and the Ca^2+^ signaling pathway. We also created the first lncRNA/circRNA-miRNA-gene interaction network based on the differentially expressed transcripts from muscle tissues, where some networks, such as circ_0017336-miR-23a-FBXL5, may be crucial in the control of muscle fiber creation. Our findings have established future investigations on the molecular mechanisms underlying muscle fiber development by identifying possible regulators and molecular regulatory networks that may be connected to the production of muscle fibers in sheep.

## Data Availability

The datasets presented in this study can be found in online repositories. The names of the repository/repositories and accession number(s) can be found in the article/Supplementary Material.

## Abbreviations

ceRNA, competing endogenous RNA; DE, endogenoustially expressed genes; DEMs, Differentially expressed miRNAs; DELs, Differentially expressed lncRNAs; DECs, Differentially expressed circRNAs; GO, Gene Ontology; KEGG, Kyoto Encyclopedia of Genes and Genomes; qPCR, quantitative Polymerase Chain Reaction; ACACB, acetyl-CoA carboxylase beta; ATP6V0A1, ATPase H+ transporting V0 subunit a1; ASAH1, N-acylsphingosine amidohydrolase 1; EFHB, EF-hand domain family member B; MYL3, myosin light chain 3; FBXL5, F-box and leucine rileucine-richotein 5; SFSWAP, splicing factor SWAP; C1QTNF7, C1q and TNF relateTNF-rerelatedong non-coding RNAs; mRNAs, messenger RNAs; miRNAs, microRNAs; circRNAs, circular RNA; MyH7, myosin heavy chain 7; MyHC, myosin heavy chain, cardiac muscle complex.
